# Effects of the Flying Start on Estimated Short Sprint Profiles Using Timing Gates

**DOI:** 10.3390/s24092894

**Published:** 2024-05-01

**Authors:** Mladen Jovanović, Dimitrije Cabarkapa, Håkan Andersson, Dora Nagy, Nenad Trunic, Vladimir Bankovic, Aleksandar Zivkovic, Richard Repasi, Sandor Safar, Laszlo Ratgeber

**Affiliations:** 1 Faculty of Sport and Physical Education, University of Belgrade, 11000 Belgrade, Serbia; 2 Jayhawk Athletic Performance Laboratory—Wu Tsai Human Performance Alliance, Department of Health, Sport and Exercise Sciences, University of Kansas, Lawrence, KS 66045, USA; 3 High Performance Center, 35246 Växjö, Sweden; 4 Faculty of Health Sciences, Doctoral School of Health Sciences, University of Pécs, 7621 Pécs, Hungary; 5 Faculty of Health Sciences, Institute of Physiotherapy and Sport Science, University of Pécs, 7621 Pécs, Hungary; 6 Faculty of Physical Culture and Sports Management, Singidunum University, 11000 Belgrade, Serbia; 7 Center for Basketball Methodology and Education, 7621 Pécs, Hungary; 8 University of Physical Education—Institute of Sport, Training Theory and Methodology Research Center, 1123 Budapest, Hungary; 9 University of Physical Education—Institute of Sport, Department of Sport Games, 1123 Budapest, Hungary

**Keywords:** sport, radar, testing, athlete, basketball, performance, speed, acceleration, power

## Abstract

Short sprints are predominantly assessed using timing gates and analyzed through parameters of the mono-exponential equation, including estimated maximal sprinting speed (MSS) and relative acceleration (TAU), derived maximum acceleration (MAC), and relative propulsive maximal power (PMAX), further referred to as the No Correction model. However, the frequently recommended flying start technique introduces a bias during parameter estimation. To correct this, two additional models (Estimated TC and Estimated FD) were proposed. To estimate model precision and sensitivity to detect the change, 31 basketball players executed multiple 30 m sprints. Athlete performance was simultaneously measured by a laser gun and timing gates positioned at 5, 10, 20, and 30 m. Short sprint parameters were estimated using a laser gun, representing the criterion measure, and five different timing gate models, representing the practical measures. Only the MSS parameter demonstrated a high agreement between the laser gun and timing gate models, using the percent mean absolute difference (%MAD) estimator (%MAD < 10%). The MSS parameter also showed the highest sensitivity, using the minimum detectable change estimator ($\%MDC_{95}$), with an estimated $\%MDC_{95}$ < 17%. Interestingly, sensitivity was the highest for the No Correction model ($\%MDC_{95}$ < 7%). All other parameters and models demonstrated an unsatisfying level of sensitivity. Thus, sports practitioners should be cautious when using timing gates to estimate maximum acceleration indices and changes in their respective levels.

## 1. Introduction

The physical attribute of sprint speed is widely recognized and esteemed in sports. Short sprints in most team sports, such as soccer, basketball, field hockey, and handball, are characterized by maximal sprinting from a stationary position over a distance that does not lead to deceleration upon completion. According to Mangine et al. [1], the highest level of anaerobic power is achieved within the initial five seconds of maximal exertion. Nevertheless, the ability to achieve maximum sprint velocity varies depending on the athlete and the sport. As per the research conducted by Ward-Smith [2], it has been observed that sprinters in track and field tend to attain their maximum speed towards the end of the race, specifically between 50 and 60 m. On the other hand, Brown et al. [3] suggest that team sports athletes reach their maximum speed much earlier in the race, typically between 30 and 40 m. The assessment of short sprint performance is commonly incorporated into a battery of physical fitness assessments for a diverse range of sports, irrespective of the dissimilarities in kinematics among athletes.

Force plates and 3D cameras are widely considered the gold-standard method for evaluating the mechanical characteristics of sprinting. However, obtaining a complete sprint profile poses practical and financial challenges [4,5]. The utilization of laboratory-grade methods, such as radar and laser technology, is a common practice in various studies [6,7,8,9]. However, these methods are generally not accessible to sports practitioners.

Undoubtedly, timing gates are the most commonly utilized approach for assessing sprint performance. It is common practice to position several gates at varying intervals to record split times, such as 10, 20, 30, and 40 m. These split times can be integrated into calculating sprint mechanical characteristics, as Morin et al. [4] and Samozino et al. [5] proposed. The utilization of estimated sprint mechanical characteristics by practitioners can serve the purpose of elucidating individual differences, quantifying the impact of training interventions, and enhancing comprehension of the constraining factors of performance, thereby conferring a benefit upon this approach.

### 1.1. Mathematical Model

The mono-exponential equation for velocity as a function of time has been employed in modeling short sprints. The concept was initially introduced by Furusawa et al. [10] and subsequently gained wider recognition through the works of Samozino et al. [5] and Clark et al. [11]. Equation (1) is utilized to determine the instantaneous horizontal velocity denoted as v, which is dependent on the time variable denoted as t, as well as two distinct model parameters.

(1) $$v\left(t\right)=MSS\times \left(1-e^{-\frac{t}{TAU}}\right)$$

The parameters of Equation (1) are the maximum sprinting speed (MSS), which is measured in meters per second (ms^−1^), and the relative acceleration (TAU), which is measured in seconds (s). The parameter TAU denotes the quotient obtained by dividing the MSS by the initial maximum acceleration (MAC), which is expressed in units of meters per second squared (ms^−2^) and can be represented by Equation (2). It should be noted that TAU represents the duration needed to attain a velocity equivalent to 63.2% of the MSS, as determined by the given Equation (1).

(2) $$MAC=\frac{MSS}{TAU}$$

While TAU is a parameter employed in the equations and subsequently estimated, it is advisable to employ and report MAC as it is more straightforward to comprehend, particularly for professionals and trainers. The equation pertaining to the horizontal acceleration, as denoted by Equation (3), can be obtained through the derivation of Equation (1).

(3) $$\left(t\right)=\frac{MSS}{TAU}\times e^{-\frac{t}{TAU}}$$

The equation for distance covered Equation (4) can be derived by means of integrating Equation (1).

(4) $$d\left(t\right)=MSS\times \left(t+TAU\times e^{-\frac{t}{TAU}}\right)-MSS\times TAU$$

**Figure 1:**
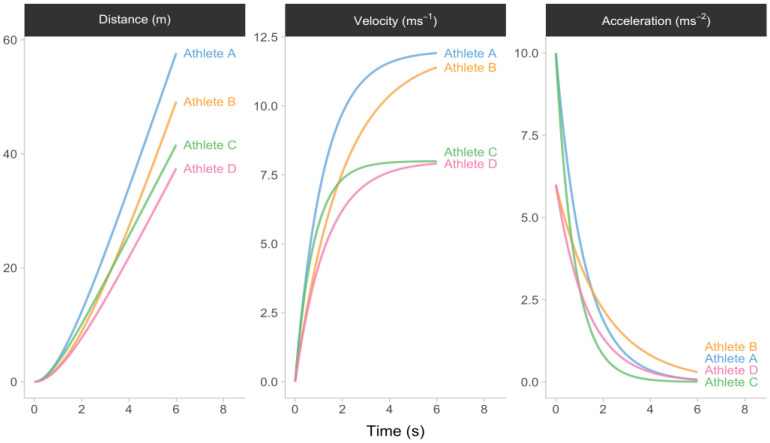
Four athletes with different maximum sprinting speed (MSS) and maximum acceleration (MAC) parameters.

**Figure 2:**
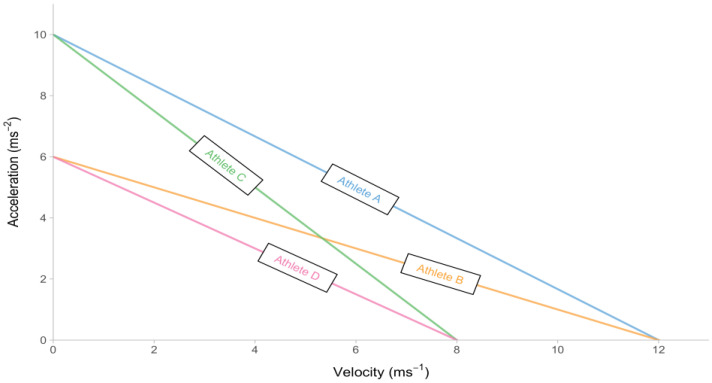
Linear relationship between velocity and acceleration, given the mono-exponential Equation (1), for four hypothetical athletes. This descriptive profile is termed acceleration–velocity profile (AVP).

Figure 1 presents a visual representation of the sprint kinematics of four hypothetical athletes who possess varying values of MSS and MAC parameters. When velocity is plotted against acceleration for the four hypothetical athletes, given Equation (1), a linear relationship can be observed, as illustrated in Figure 2. This model feature facilitates the process of simplification, which involves generating an aggregated summary of the short sprint kinematics through the utilization of two descriptive parameters, namely MSS and MAC. The nomenclature employed to describe the aforementioned relationship (Figure 2) is known as the acceleration–velocity profile (AVP).

The parameter known as maximal relative power (PMAX), which is expressed in units of Watts per kilogram (W/kg), is frequently calculated and documented in academic literature [4,5]. The calculation of PMAX is performed utilizing Equation (5). The approach employed for PMAX estimation in this context does not take into account the impact of air resistance, thereby indicating the net or relative propulsive power.

(5) $$PMAX=\frac{MSS\times MAC}{4}$$

The acceleration–velocity profile (i.e., MSS and MAC parameters) can thus serve as a model or representation of two of the three prevalent phenomenological characteristics in sprinting [12,13,14,15,16,17]. These include (1) the ability to achieve maximum forward acceleration (represented with the MAC parameter); (2) the ability to attain maximum speed (represented with the MSS parameter); and (3) the ability to sustain speed while resisting the onset of fatigue (which is not a factor in short sprint performance as there is no deceleration involved).

To demonstrate this simplification, the following 5, 10, 20, 30, and 40-m split times (1) 1.45, 2.23, 3.59, 4.88, and 6.14 s; and (2) 1.51, 2.28, 3.58, 4.77, and 5.92 s are more easily simplified by using MSS and MAC parameters. The aforementioned splits were generated using an MSS of 8 ms^−1^ and a MAC of 7 ms^−2^ for the first and an MSS of 9 ms^−1^ and a MAC of 6 ms^−2^ for the second. It would be hard to discern these traits from simply knowing the split times.

### 1.2. Model Parameters Estimation Using Laser/Radar Gun

**Figure 3:**
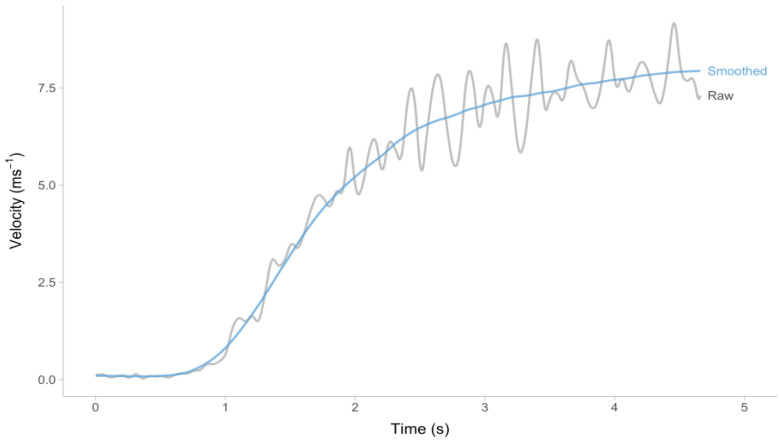
Sample laser gun (Musclelab™ LaserSpeed, Ergotest Technology AS, Langesund, Norway) output during 30 m sprint. The gray line indicates raw velocity (sampled at 1000 Hz). The blue line indicates smoothed velocity (the exact filtering/smoothing method is a proprietary secret of Ergotest Technology AS).

The problem related to estimating model parameters using a laser/radar gun can be illustrated through a simple example. The data presented in Figure 3 pertain to the velocity of a standing start 30 m short sprint over time. The data was collected using a laser gun (CMP3 Distance Sensor, Noptel Oy, Oulu, Finland) and was sampled at a rate of 2.56 KHz. A polynomial function modeling the relationship between distance and time was employed and subsequently resampled at a frequency of 1000 Hz using Musclelab™ v10.232.107.5298, a software developed by Ergotest Technology AS located in Langesund, Norway. As illustrated in Figure 3, Musclelab™ provides measurements for both unprocessed velocity (raw velocity in Figure 3), and processed velocity (smoothed velocity in Figure 3). The specific technique used for filtering and smoothing is confidential information of Ergotest Technology AS.

As evidenced by the data presented in Figure 3, the initiation of the sprint does not occur at the onset of the time interval (*t* = 0 s). Therefore, it is crucial to trim the data that come before the sprint itself. One possible approach is to apply a filter to the velocity data, specifically targeting values that exceed a predetermined threshold (e.g., 0.5 ms^−1^). Furthermore, it is imperative to adjust Equation (1) by introducing an additional parameter for estimation, namely time-correction (TC) (Equation (6)). The TC parameter functions as an intercept in the model, enabling it to make adjustments in both directions and to make predictions regarding the onset of sprinting.

(6) $$v\left(t\right)=MSS\times \left(1-e^{-\frac{t-TC}{TAU}}\right)$$

The process of determining parameters, specifically MSS, TAU, and TC, as denoted in Equation (6), is accomplished through the utilization of non-linear least squares regression. Researchers, coaches, and sports scientists have utilized the built-in solver function of Microsoft Excel (Version 16.0, Microsoft Corporation, Redmond, WA, USA) to conduct short sprint modeling [4,5,11,18,19,20]. The open-source package {shorts} for the R-language [21] has recently incorporated various functionalities, along with supplementary features [22,23]. The package employs the nlsLM function from the {minpack.lm} package [24] for estimating model parameters using non-linear least squares regression. In contrast to the solver function integrated within Microsoft Excel, the {shorts} package offers a more feature-rich, flexible, transparent, and reproducible environment framework for constructing models of short sprints. Accordingly, this study will employ the {shorts} package to compute model parameters.

**Figure 4:**
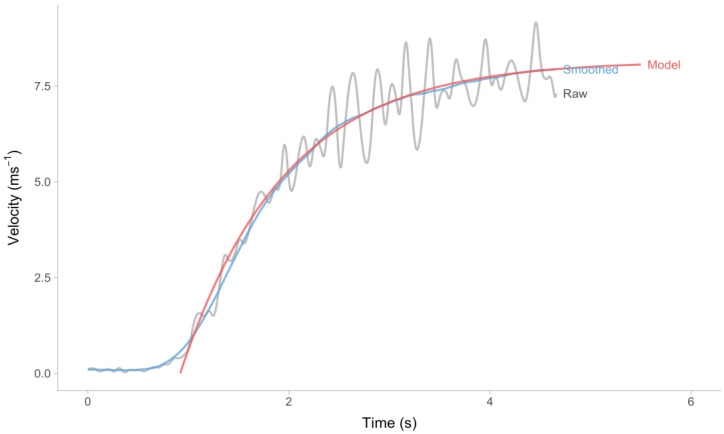
Modified mono-exponential model (Equation (6)) applied to the laser gun sample’s raw velocity (Figure 3). The gray line indicates raw velocity (1000 Hz). The blue line indicates smoothed velocity (the exact filtering method is a proprietary secret of Ergotest Technology AS). The red line represents mono-exponential model prediction.

The estimated MSS, TAU, MAC, and TC parameter values were obtained from the sample provided in Figure 3. The estimated values for MSS, TAU, MAC, and TC were found to be 8.16 ms^−1^, 1.03 s, 7.9 ms^−2^, and −0.92 s, respectively. Figure 4 illustrates the adjusted mono-exponential model’s (Equation (6)) predictions (indicated by the red line) in comparison to the data collected from the laser gun.

**Figure 5:**
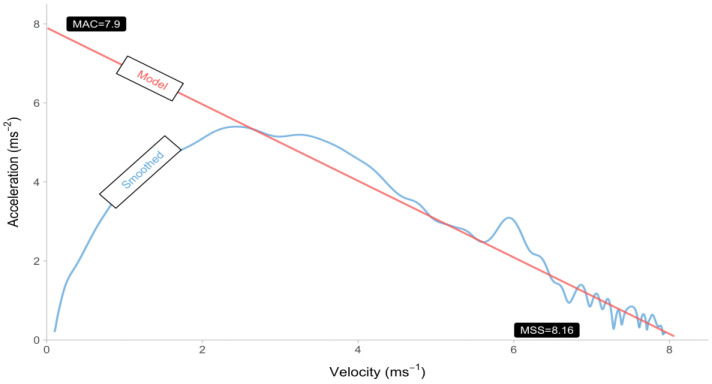
Acceleration–velocity trace using smoothed and model-predicted velocities from Figure 4. Acceleration for every sample is estimated using a = Δv/Δt. Estimated maximum sprinting speed (MSS) and maximum acceleration (MAC) parameters are written close to the *x*- and *y*-axes.

The computation of acceleration can be achieved through the utilization of both smoothed and model-predicted velocity, employing the a = Δ*v*/Δ*t* methodology. Figure 5 depicts the relationship between velocity and acceleration through the utilization of both smoothed and model-predicted velocity. Figure 5 illustrates a notable inconsistency between the smoothed and model-predicted data. This discrepancy arises due to the assumption made by the mono-exponential model, which considers the maximum acceleration to occur when the velocity is zero. The utilization of a standing start instead of a block start is the likely reason for the deviation from the aforementioned assumption in the method that employs smoothed observed velocity.

It is important to emphasize that all three velocities (i.e., raw, smoothed, and model-predicted) represent approximations of much more complex short sprint performance. This aligns with the concepts of *Small* and *Large World*, in which the Small World refers to the self-contained and internally consistent world of a given model, whereas the Large World pertains to the wider context in which the model is applied [25,26,27,28,29]. Raw velocity is the velocity estimated from the body point closest to the laser gun, and that is often the low back of the athlete since the laser was positioned at approximately 1 m from the ground. As previously stated in the Introduction, force plates and 3D cameras are widely recognized as the preferred methods for evaluating the mechanical characteristics of sprinting, specifically in terms of estimating the velocity of the center-of-mass (COM). Therefore, radar and laser may be regarded as the silver standard, while the raw velocity can be seen as the most accurate estimate of the COM velocity.

The velocity that has been smoothed refers to the velocity that has been averaged over steps (i.e., step-averaged velocity), without taking into account the acceleration and deceleration that is evident in the raw velocity as depicted in Figure 3. The purpose of this smoothing is to simplify the analysis of kinematics. The mono-exponential model, encompassing both Equations (1) and (6), provides a simplified approach to analyzing short sprint kinematic performance. This model utilizes two variables, namely MSS and MAC, to describe and consolidate the short sprint performance. The simplification in question is highly practical, as it facilitates the comparison of athletes and enables the monitoring of changes in training interventions. However, it is important to note that this approach may yield misleading results, as illustrated in Figure 5, due to potential disparities between the smoothed velocity and model predictions.

### 1.3. Estimation of Model Parameters Using Timing Gate Split Times

The dataset in Table 1 includes a sample of split times that were recorded during a sprint performance of 40 m. The split timings were measured using timing gates placed at a distance of 5, 10, 20, 30, and 40 m.

The procedure for estimating model parameters using split times entails employing distance as an independent variable (i.e., predictor) and time as the dependent variable (i.e., outcome). As a result, Equation (4) is structured in the form of Equation (7).

(7) $$t\left(d\right)=TAU\times W\left(-e^{\frac{-d}{MSS\times TAU}}-1\right)+\frac{d}{MSS}+TAU$$

The symbol W appearing in Equation (7) denotes the mathematical function known as Lambert’s W function. This function is characterized as the inverse of the multivalued function f(w) = w*e*^w^ [30,31]. The use of Equation (4), where time serves as the independent variable and distance as the dependent variable, is a prevalent practice in academic research [18,20]. It is advisable to refrain from utilizing this approach as it has the potential to generate bias in the estimated parameters [32] (p. 341). While the bias in question may not have practical significance when it comes to profiling short sprints, it is a flawed statistical practice and should be eschewed. Therefore, it is advisable to employ the statistically accurate Equation (7) for the estimation of model MSS and TAU parameters.

**Figure 6:**
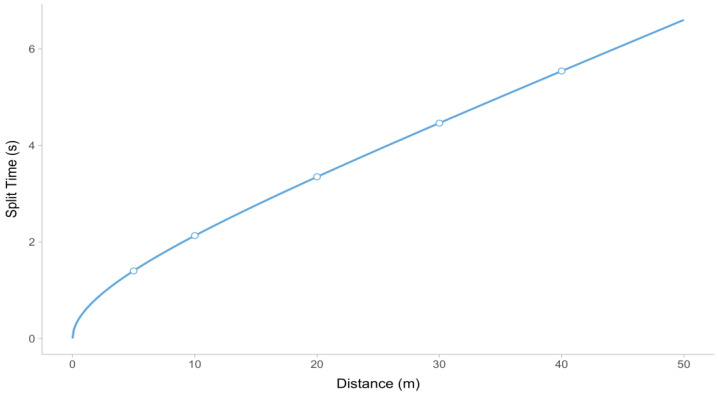
Split time predictions using the Equation (7) model (depicted as a line) and observed timing gate split times from Table 1 (depicted as points).

Based on the split times provided in Table 1, the estimated values for MSS, TAU, and MAC parameters are 9.54 ms^−1^, 1.37 s, and 6.96 ms^−2^, respectively. Figure 6 illustrates the predictions of the Equation (7) model.

### 1.4. Inaccuracies in Estimated Parameters Using Timing Gates Due to Flying Start and Reaction Time

To obtain accurate estimates of the short sprint parameters, it is crucial to synchronize the initiation of force generation with the commencement of the sprint timing, usually known as the “first movement” trigger. This has been highlighted in various studies [5,33,34,35,36,37]. The acquisition of sprint data through timing gates poses a challenge that can significantly affect the estimated parameters.

In order to illustrate the effect, consider a hypothetical scenario involving three triplet siblings, Andrew, Ben, and Cole, who possess identical characteristics for short sprints, including an MSS of 9.5 ms^−1^, TAU of 1.357 s, MAC of 7 ms^−2^, and PMAX of 16.625 W/kg (which are indicative of authentic or true short sprint parameters). All three triplet siblings executed a sprint of 40 m from a stationary position, with timing gates placed at distances of 5, 10, 20, 30, and 40 m. Andrew and Ben activate the timing system when they cross the beam at the beginning of the sprint (i.e., *d* = 0 m). For Cole, the timing system is activated after the gunfire. Andrew embodies the theoretical framework positing that the commencement of force production and the initiation of timing are in complete synchrony. The split times belonging to Andrew have already been employed in Table 1.

Conversely, Ben elects to displace himself marginally from the primary timing gate (i.e., for a flying distance of 0.5 m) and employs body rocking to instigate the sprint commencement. To clarify, Ben employs a technique known as a flying start, frequently utilized when testing athletes in field sports. The utilization of a flying start distance is frequently suggested from a measurement standpoint to prevent untimely activation of the timing system caused by elevated knees or swinging arms. This recommendation is supported by various studies [35,37,38,39,40]. A flying start at the beginning of a short sprint can also be attributed to the act of body rocking during the initial standing start. It is evident that any commencement characterized by a disparity between the initial force production and the onset time has the potential to result in distorted estimated parameters. The difficulty in enhancing sprint characteristics coupled with inconsistent starts can potentially mask the impact of the training intervention or, in other words, reduce the sensitivity of the measurement to detect true changes.

**Figure 7:**
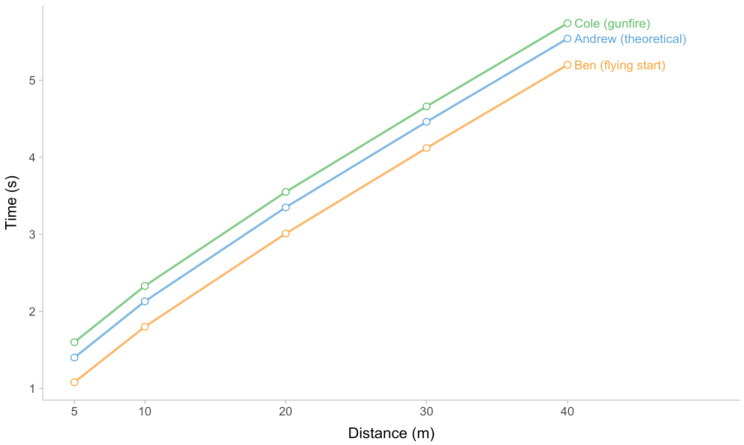
Andrew, Ben, and Cole recorded their respective split timings while running 40 m. The sprint performances of the three brothers are indistinguishable, although they employ distinct sprint beginnings, leading to variations in their split timings.

Cole’s start is set off by gunfire. Therefore, his split times include an extra response time of 0.2 s. This situation resembles a hypothetical circumstance in which an athlete inadvertently activates a timing mechanism by standing close to the initial timing gate. The data provided by Cole can be utilized to illustrate the impact of this situation on the estimated parameters. In this hypothetical scenario, utilized timing gates offer high accuracy, with measurements being recorded up to two decimal places (specifically, the nearest ten milliseconds). However, it is essential to note that this numeric precision also introduces a potential source of inaccuracy in the measurements obtained. The sprint splits are visually depicted in Figure 7.

The outcomes presented in Table 2 indicate that the estimated short sprint parameters for each of the three siblings deviate from the true parameters employed to produce the data, which represent their genuine short sprint characteristics. The estimated parameters of all three siblings are subject to bias owing to the precision of the timing gates, which is limited to two decimal places (i.e., 10 ms). The presence of bias in the estimated parameters for Ben can be attributed to the inclusion of a flying start, whereas for Cole, the bias can be attributed to the involvement of reaction time in the split times.

### 1.5. How to Overcome Bias in Estimated Parameters When Using Timing Gates?

According to the existing literature, a feasible approach to convert to “first movement” triggering while employing the suggested 0.5 m flying distance behind the initial timing gate is to apply a correction factor of +0.5 s (i.e., the addition of +0.5 s to split times) [33,34,35,36]. The study conducted by Haugen et al. [33] revealed a noteworthy finding that the mean disparity in the 40 m sprint time between the standing start initiated by a photocell trigger and a block start to gunfire was 0.27 s. As a result, it is imperative to incorporate a timing correction factor to avoid any additional imprecision in the evaluation of mechanical parameters. However, if the correction factor is too large or small, it may also lead to imprecision in the mechanical parameters.

### 1.6. Estimated Time Correction Model

Rather than relying on a priori time correction values from the existing literature, it is possible to estimate this parameter by utilizing the provided data in conjunction with MSS and TAU. Stenroth et al.’s [19] study on sprint profiling in ice hockey suggests utilizing a comparable methodology, referred to as the time shift method, and an estimated parameter termed the time shift parameter. Consistent with existing literature and utilizing the adjusted mono-exponential equation employed for laser gun data modeling (Equation (6)), the present study designates this parameter as time correction (TC). Implementing the TC parameter in the original Equation (7) now yields the new Equation (8).

(8) $$t\left(d\right)=TAU\times W\left(-e^{\frac{-d}{MSS\times TAU}}-1\right)+\frac{d}{MSS}+TAU+TC$$

Equation (8) is utilized as the model definition in the estimated time correction (Estimated TC) model. The model using Equation (7) is termed the No Correction model throughout this study. Models in which TC is constant (i.e., by simply adding predefined TC to split times) are termed fixed time correction (Fixed TC) models.

From a regression standpoint, the TC parameter can be interpreted as an intercept. Assuming a fixed time shift is present, such as in the case of reaction time or premature triggering of timing equipment, the TC parameter can be beneficial in unbiasing estimated parameters (i.e., MSS and TAU). Comparing Andrew and Cole as presented in Figure 7, it can be observed that the lines representing their respective split times exhibit a parallel relationship. The utilization of the Estimated TC model in this particular scenario has the potential to mitigate bias that may exist between Andrew and Cole. In Ben’s case, the utilization of the Estimated TC model has the potential to alleviate bias in estimated parameters as well. However, upon closer examination of Figure 7, it becomes apparent that the lines representing Ben and Andrew are non-parallel. The non-constant time shift is attributed to the pre-existing velocity at the triggering of the initial timing gate. Thus, the inclusion of the TC parameter will not completely remove the bias in Ben’s case.

The aforementioned models, namely the Fixed TC models with values of +0.3 and +0.5 s, as well as the Estimated TC model, were utilized to analyze the split times of Andrew, Ben, and Cole. The model parameters that were estimated can be located in Table 3, alongside the parameter values that were previously estimated using the No Correction model. As evidenced by the data presented in Table 3, the inclusion of a +0.3 s value yielded favorable results for Ben in terms of approximating the true parameter values. Conversely, the incorporation of a +0.5 s value had an adverse effect on the unbiased estimation of parameters. The Estimated TC model demonstrated efficacy in mitigating bias in parameter estimates across all three brothers. The estimated TC parameter for Cole exhibited a high degree of proximity to the actual reaction time of 0.2 s.

### 1.7. Estimated Flying Distance Model

The Estimated TC model demonstrated a favorable performance in Ben’s case (sibling involving a flying start). However, rather than relying on the assumption of constant time shift to mitigate bias in the estimates, an alternative approach involves incorporating the flying start distance (FD) into the model definition as an additional parameter. Incorporating the FD parameter into Equation (7) yields Equation (9).

(9) $$\begin{array}{ll} t\left(d\right) & =\left(TAU\times W\left(-e^{\frac{-(d+FD)}{MSS\times TAU}}-1\right)+\frac{d+FD}{MSS}+TAU\right)\\ & -\left(TAU\times W\left(-e^{\frac{-FD}{MSS\times TAU}}-1\right)+\frac{FD}{MSS}+TAU\right) \end{array}$$

Similar to the Fixed TC and Estimated TC models, the FD parameter has the option to be either estimated or fixed. If the flying start distance is a known value (e.g., 0.5 m), it can be utilized as a constant parameter. The model that utilizes a fixed FD parameter value is denoted as a fixed flying start distance (Fixed FD) model. On the other hand, the model in which the FD parameter is estimated together with MSS and TAU parameters is denoted as the estimated flying star distance (Estimated FD) model.

**Figure 8:**
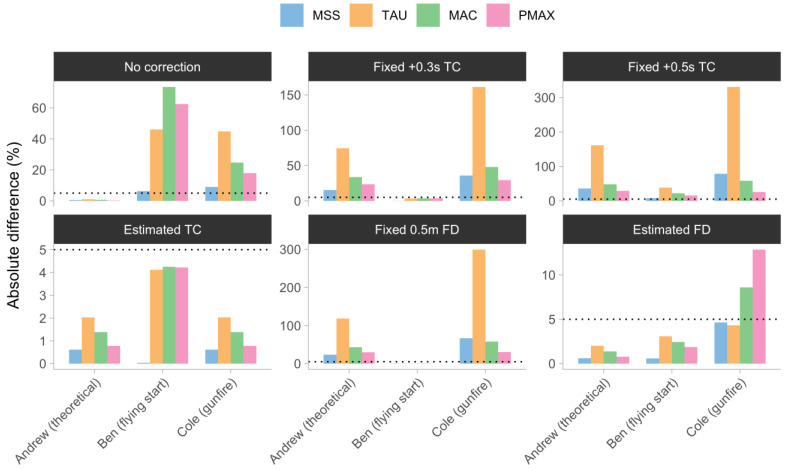
Estimated sprint parameters for Andrew, Ben, and Cole using (1) No Correction; (2) Fixed +0.3 s time correction (Fixed +0.3 s TC); (3) Fixed +0.5 s time correction (Fixed +0.5 s TC); (4) Estimated time correction (Estimated TC); (5) Fixed 0.5 m flying start distance (Fixed 0.5 m FD); and (6) Estimated flying start distance (Estimated FD) models expressed as absolute percent difference from the true parameter value. Dotted horizontal lines represent a 5% absolute difference used as a visual anchor. Note: MSS—maximum sprinting speed (expressed ms^−1^). TAU—relative acceleration (expressed in seconds); MAC—maximum acceleration (expressed in ms^−1^), PMAX—maximal relative power (expressed in W/kg); dotted horizontal line—visual anchor using fixed 5% absolute percent difference, used for easier visual comparison between models.

Table 3 encompasses the complete set of model estimates for a trio of siblings, comprising both the Fixed 0.5 m FD and Estimated FD models. A visual depiction in the form of Figure 8 accompanies Table 3. In order to standardize the comparison of estimates, the absolute percent difference from the true parameter value is employed. A visual anchor is employed in the form of a fixed 5% absolute percent difference, represented by a dotted horizontal line in Figure 8, to facilitate visual comparison among the models.

The No Correction model generated parameters that were biased toward Ben and Cole. The Fixed +0.3 s TC model produced unbiased parameters for Ben, but resulted in a greater degree of parameter bias for Andrew and Cole. The introduction of a fixed time correction of +0.5 s in the Fixed +0.3 s TC model resulted in a significant bias for all three siblings. The Estimated TC and Estimated FD models exhibited minimal bias for Andrew, whereas they effectively rectified the model parameters for Ben and Cole. The model parameters for Ben were successfully adjusted to eliminate bias using the Fixed 0.5 m FD model. However, this resulted in a significant bias for Andrew and Cole. In general, the parameter that exhibited the least amount of bias was MSS. This suggests that, in the context of this uncomplicated simulation, MSS is the most resilient parameter among the four.

It is important to acknowledge that every model definition incorporates a specific assumption regarding the mechanism of data generation (i.e., data-generating process, or DGP). The No Correction model postulates the ideal alignment of sprint initiation with the commencement of timing. The Estimated TC model incorporates a basic intercept that can facilitate the estimation of parameters in situations where a time shift is presumed to be present and constant, such as when reaction time is a factor or when the initial timing gate is triggered prematurely. The utilization of the Estimated TC model is also applicable in scenarios where a “flying start” is employed. However, it presupposes a constant time shift, which is not the case in such situations due to the velocity already acquired at the start. The Fixed FD and Estimated FD models presuppose the presence of a flying sprint in the data-generating process. As evidenced by the estimates presented in Table 3, these models may be ill-defined in cases where there is no flying distance component, but a temporal displacement is present. Each of the three models postulates that athletes undergo acceleration in accordance with Equation (1). As demonstrated in Figure 5, this is not necessarily the case.

The No Correction model is a widely utilized approach for estimating short sprint parameters, whereas the Estimated TC and Estimated FD models are novel model definitions that require additional scientific validation for their application.

The effect of starting position on the short sprint modeling using timing gates represents a practical problem for practitioners and researchers. Elimination of the bias in estimated parameters introduced due to the flying start is imperative to enhance the validity of short sprint profiles and to improve their sensitivity to intervention changes.

A recently published study by Jovanović [41] explored the behavior of the No Correction, Estimated TC, and Estimated FD mono-exponential models under simulated conditions using a varying flying start distance, from 0 to 0.5 m. This work involved simulation of the timing gate split times positioned at 5, 10, 20, 30, and 40 m, with a varying flying start distance and rounding to the closest 10 ms under known (i.e., true) MSS and MAC parameters. The estimated parameters (i.e., MSS, TAU, MAC, and PMAX), using the No Correction, Estimated TC, and Estimated FD models, were compared against the true parameters (i.e., parameters used to simulate the timing gate split times) using the distribution of the percent difference, as well as the minimum detectable change estimator. The results of this simulation study demonstrated bias in estimating short sprint parameters using the No Correction model, negligible bias for the Estimated TC model, and no bias for the Estimated FD model. The unexpected finding of this study was that the No Correction model sensitivity to detect changes in MAC and TAU parameters outperformed the other two models.

The major limitation of the study by Jovanović [41] was that it involved theoretical simulation, rather than the athletes. The current study aims to add to the findings by estimating validity and sensitivity of the No Correction, Fixed TC, Estimated TC, Fixed FD, and Estimated FD mono-exponential models for estimating short sprint parameters (i.e., MSS, TAU, MAC, and PMAX) using timing gates and a fixed flying start distance (0.5 m) against the criterion measure (i.e., the laser gun). We hypothesized that the No Correction model will induce bias in short sprint parameters and that the Estimated TC and Estimated FD models will alleviate this bias and improve the sensitivity of short sprint profiling to detect the true change in individual sprint characteristics.

## 2. Materials and Methods

### 2.1. Experimental Approach

This study involves the assessment of athletes’ sprinting performance over a distance of 30 m, commencing from a stationary position, 0.5 m behind the initial timing gate. The measurement of their performance was conducted through the use of a laser gun and timing gates. Since the true individual parameters are unknown, laser gun estimates served as the criterion measure used to compare and evaluate the timing gates estimates. In addition to estimating the agreement of the timing gates and laser gun, the sensitivity of the measures to detect changes in parameters was also established.

### 2.2. Participants

This part of the study involved the participation of 31 basketball players, comprising 23 males (age of 16.1 ± 1.0 years, height of 188.3 ± 7.5 cm, and body mass of 69.5 ± 10.8 kg) and 8 females (age of 16.1 ± 1.4 years, height of 170.5 ± 7.5 cm, and body mass of 60.9 ± 7.6 kg). These players were selected from the highest youth level in Hungary. The participants were duly apprised of the potential hazards and advantages of their involvement in the study, and a written authorization was procured from both the participants and their parents. The research adhered to the ethical guidelines approved by the Faculty of Sport and Physical Education at the University of Belgrade, Serbia (02-877/23-2, 9 May 2023), and was conducted in accordance with the most recent version of the Declaration of Helsinki.

### 2.3. Procedures

**Figure 9:**
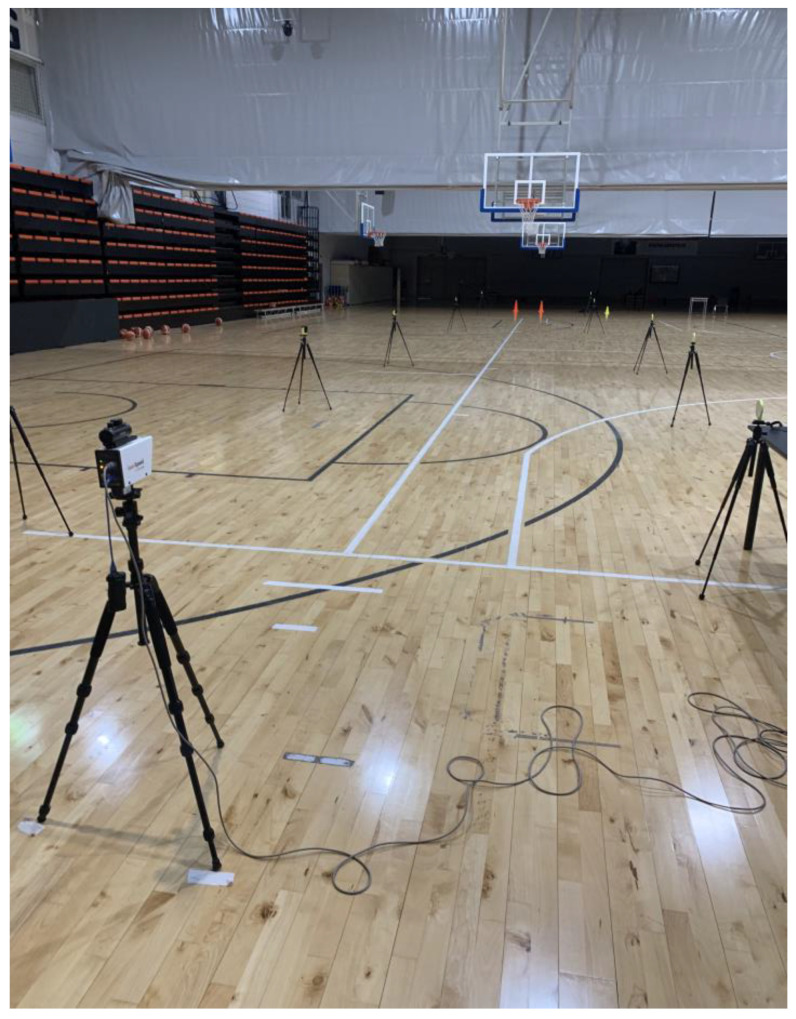
Laser gun and timing gates setup.

Prior to evaluating sprint performance, a standardized warm-up protocol lasting 15 min was executed. The warm-up involved a series of mobility and running exercises performed repeatedly within a 20 m distance, culminating in three incremental sub-maximal sprints covering a distance of 30 m. Following the warm-up, the participants executed two trials of maximal sprints covering a distance of 30 m, with a minimum rest period of 3 min between each trial. If equipment failure occurred, an additional sprint was executed as necessary. The sprint times were recorded using a set of five wireless photocell pairs (WittyGATE™ v1.5.34, Microgate S.r.l, Bolzano, Italy) positioned at the start line, as well as at distances of 5, 10, 20, and 30 m (Figure 9). The accuracy of the timing measurements was 0.01 s. At the beginning of each sprint, the participants assumed a split stance with their lead foot positioned behind a line affixed to the floor at a distance of 0.5 m from the initial pair of photocells. The photocells were situated at a height of 1 m to prevent premature interruption of the beam by the upper body during the starting position. The velocity measurements were continuously recorded for each attempt utilizing a laser gun (CMP3 Distance Sensor, Noptel Oy, Oulu, Finland) at a sampling rate of 2.56 KHz. A polynomial function was utilized to model the relationship between distance and time, which was subsequently resampled at a frequency of 1000 Hz through the use of Musclelab™ v10.232.107.5298 (Ergotest Technology AS, Langesund, Norway). The laser gun was situated at a distance of roughly 3 m from the initial timing gate, while the reference point (i.e., zero distance) was established at a distance of 1 m behind the initial timing gate (Figure 9). The entirety of the sprints were executed within the confines of an indoor basketball facility.

To compare the short sprint mechanical parameters, (1) the maximal sprinting speed (MSS); (2) the relative acceleration (TAU); (3) the maximal acceleration (MAC); and (4) the net relative propulsive power (PMAX) were calculated based upon the sprint times at 5, 10, 20, and 30 m measured with the timing gates and with the laser system by using open-source {shorts} package [22,23]. The mechanical parameters for the timing gates were estimated through five different models: (1) No Correction; (2) Fixed +0.3 s time correction (Fixed +0.3 s TC); (3) Estimated time correction (Estimated TC); (4) Fixed 0.5 m flying start distance (Fixed 0.5 m FD); and (5) Estimated flying start distance (Estimated FD) models explained previously. Sprint mechanical parameters for the laser gun were estimated using the raw velocity–time signal and time correction polynomial model (Equation (6)), after filtering out velocities below 0.5 ms^−1^ using the smoothed velocity provided by the Musclelab™ software.

The inclusion criteria for the sprint trials encompassed both timing gates and laser gun data. The study excluded trials that exhibited deceleration in timing gate split times, wherein the mean velocity of a particular split was slower than that of the preceding split. Furthermore, laser gun trials that exhibited a trace length of less than 29 m were excluded from subsequent analysis. In addition to the observed timing gate split times, simulated splits times were produced and utilized in the analysis, representing the expected results, as explained by Jovanović [41]. Simulated timing gate split times were generated using laser estimates as a generative model, assuming a 0.5 m flying distance, and closest 0.01 s rounding.

### 2.4. Statistical Analysis

Agreements between short sprint parameter estimates using the laser gun and timing gates were estimated using the percent difference (%Diff) estimator (Equation (10)), which was calculated for every athlete and trial.

(10) $$\%Diff=100\times \frac{\left(Timing\;Gates-Laser\right)}{Laser}$$

Using individual percent difference scores, the percent bias (%Bias, or mean percent difference; Equation (11)) and percent mean absolute difference (%MAD; Equation (12)) were calculated.

(11) $$\%Bias=\frac{1}{N}\sum_{i=1}^{N}\left(\%Diff_{i}\right)$$

(12) $$\%MAD=\frac{1}{N}\sum_{i=1}^{N}\left|\%Diff_{i}-\bar{\%Diff}\right|$$

Statistical inferences for the %Bias and %MAD estimators were provided using the 5000 resamples bootstrap and 95% bias-corrected and accelerated (BCa) confidence intervals [42,43,44,45].

Practitioners are frequently concerned about whether they may utilize estimated parameter values to monitor changes in the true parameters in addition to estimating the agreement between them. Thus, an estimate of the sensitivity represents crucial information to decide whether a given measure can be practically used to monitor changes. A minimal detectable change estimator with a 95% confidence (%MDC_95_) [43,46] was utilized to estimate this sensitivity. The %MDC_95_ value might be regarded as the minimum amount of change that needs to be observed in the estimated parameter for it to be considered a true change.

The sensitivity of the timing gates to detect changes in parameters, estimated using agreement with the laser gun, assumes that there is no random error in the laser gun estimates. In other words, this method assumes that the laser gun estimates represent the true parameter value.

The percent residual standard error (%RSE) of the pooled (i.e., Trial 1 and Trial 2) linear regression between the laser gun (predictor) and timing gates (outcome) (Equation (13)) was utilized to calculate %MDC_95_ (Equation (14)) for short sprint parameters. Assuming no random error involved in the laser gun estimates, %RSE represents the percent standard error of the measurement (%SEM) in the timing gates estimates.

(13) $$\%RSE=\sqrt{\frac{\sum_{i=1}^{N}{\left(100\times \frac{y_{i}-\hat{y_{i}}}{\hat{y_{i}}}\right)}^{2}}{N-2}}$$

(14) $$\%MDC_{95}=\%RSE\times \sqrt{2}\times 1.96$$

Statistical inference for the %MDC_95_ estimator was provided using the 5000 resamples bootstrap and 95% bias-corrected and accelerated (BCa) confidence intervals [42,43,44,45].

## 3. Results

The dataset in Table 4 comprises the total count of trials that were subjected to subsequent analysis following the exclusion of trials that did not satisfy the established inclusion criteria. The Estimated FD model could not be fitted for specific athletes in Trial 1, as denoted in Table 4.

The measured timing gate split times for 5, 10, 20, and 30 m marks ranged from 0.9 to 1.41, 1.58 to 2.26, 2.78 to 3.69, and 3.95 to 5.11 s, respectively. The calculated average split velocities for 0–5 m ranged from 3.55 to 5.56, for 5–10 m from 3.55 to 5.56, for 10–20 m from 3.55 to 5.56, and for 20–30 m from 3.55 to 5.56 ms^−1^. The estimated individual parameter values across Trial 1 and Trial 2 for the laser gun, No Correction, Fixed +0.3 s TC, Estimated TC, Fixed 0.5 m FD, and Estimated FD models ranged from 6.6 to 9.68 ms^−1^ for MSS, from 0.36 to 2.13 s for TAU, from 4.18 to 23.17 ms^−2^ for MAC, and from 8.49 to 48.2 W/kg for the PMAX parameter.

The pooled (i.e., Trial 1 and Trial 2 combined) individual parameter agreement using the percent difference (%Diff) between the laser gun and timing gates estimates for the No Correction model ranged from −69 to 196.3%, for the Fixed +0.3 s TC model ranged from −34.2 to 66.2%, for the Estimated TC model ranged from −44.9 to 72.5%, for the Fixed 0.5 m FD model ranged from −41.6 to 91.3%, and for the Estimated FD model ranged from −41.3 to 85.4%.

The estimated mean percent difference (%Bias) between the laser gun and timing gates parameter estimates, using pooled Trial 1 and Trial 2 data for the No Correction model ranged from −46.1 to 88.5%, for the Fixed +0.3 s TC model ranged from 0.9 to 3.2%, for the Estimated TC model ranged from −10.9 to 14.8%, for the Fixed 0.5 m FD model ranged from 1.3 to 7.7%, and for the Estimated FD model ranged from −0.3 to 5.8%.

The MSS parameter demonstrated the lowest bias across all timing gate models, ranging from −5.4 to 1.3%, while the MAC parameter demonstrated the highest bias ranging from 1.3 to 88.5%.

**Figure 10:**
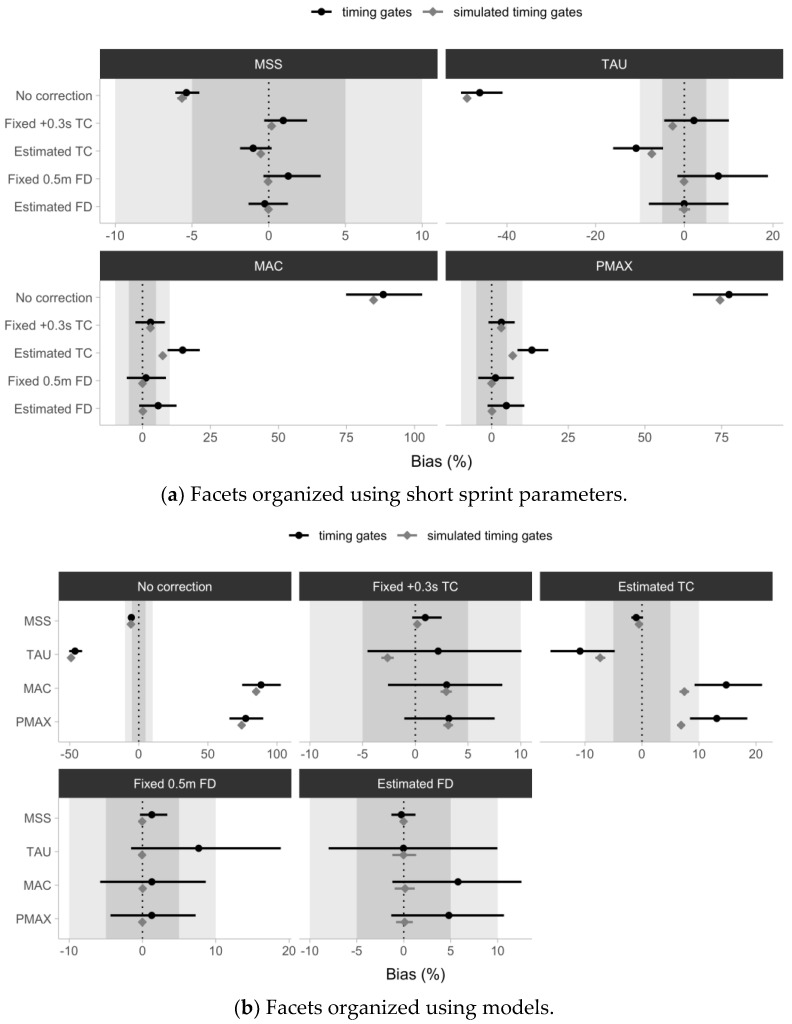
Estimated short sprint parameters percent bias (i.e., mean difference) between the laser and (1) No Correction; (2) fixed +0.3 s time correction (Fixed +0.3 s TC); (3) Estimated time correction (Estimated TC); (4) Fixed 0.5 m flying start distance (Fixed 0.5 m FD); and (5) Estimated flying start distance (Estimated FD) models, for both observed timing gate split times (black ●) and simulated timing gate split times (gray ◆). Simulated timing gate split times are generated using laser estimates as a generative model, assuming a 0.5 m flying distance, and 0.01 s time rounding. Simulated timing gates models thus represent expected bias, given theoretical assumptions. Pooled data sets (i.e., Trial and Trial 2) were utilized. Gray bars represent a ±5 and ±10% difference used as visual anchors. Error bars represent 95% bias-corrected and accelerated (BCa) 5000 resamples bootstrap confidence intervals. Note: MSS—maximum sprinting speed (expressed in ms^−1^). TAU—relative acceleration (expressed in seconds); MAC—maximum acceleration (expressed in ms^−2^); PMAX—maximal relative power (expressed in W/kg).

Figure 10 depicts the estimated %Bias and accompanying 95% confidence intervals as error bars. Visual inspection of Figure 10 demonstrates that (1) simulated timing gates and observed data confidence intervals overlap or touch for all models (apart from the Estimated TC model for the MAC and PMAX parameters); (2) the No Correction model confidence intervals excluded the zero line for all parameters; and (3) the Estimated TC model confidence intervals excluded the zero line for all parameters except MSS.

The estimated mean percent absolute difference (%MAD) between the laser gun and timing gates parameter estimates, using pooled Trial 1 and Trial 2 data for the No Correction model ranged from 5.4 to 88.5%, for the Fixed +0.3 s TC model ranged from 3.5 to 19.3%, for the Estimated TC model ranged from 2.7 to 19.9%, for the Fixed 0.5 m FD model ranged from 4.5 to 26.2%, and for the Estimated FD model ranged from 3 to 22.3%.

The MSS parameter demonstrated the lowest %MAD across all timing gate models, ranging from 2.7 to 5.4%, while the MAC parameter demonstrated the highest %MAD ranging from 15.3 to 88.5%.

**Figure 11:**
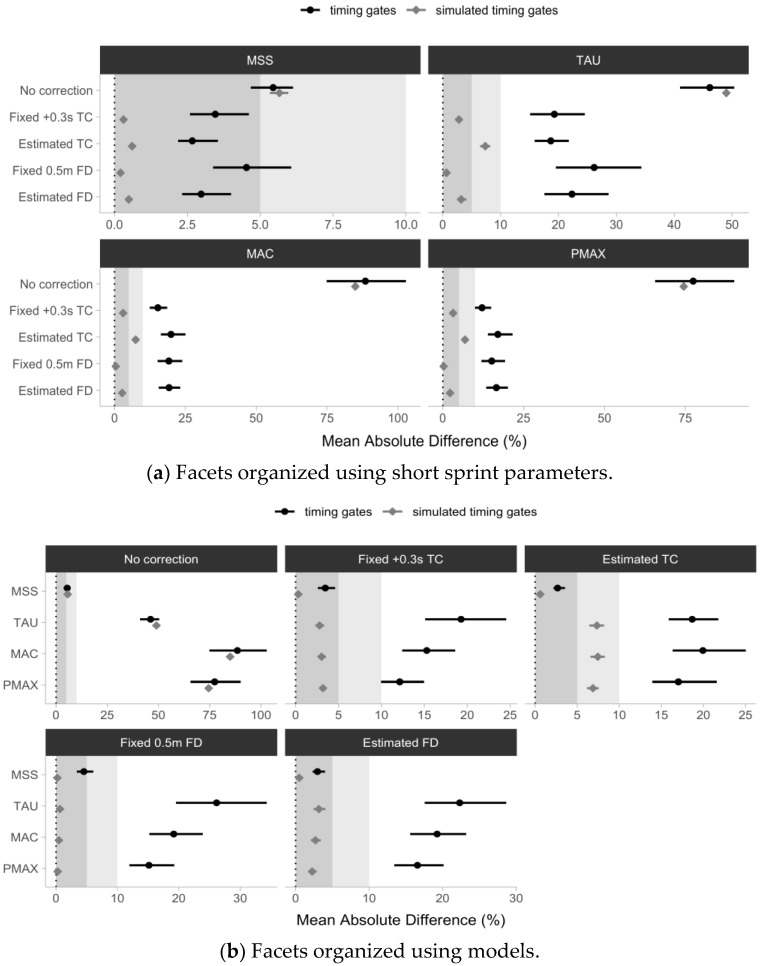
Estimated short sprint parameters percent mean absolute difference (%MAD) between the laser and (1) No Correction; (2) Fixed +0.3 s time correction (Fixed +0.3 s TC); (3) Estimated time correction (Estimated TC); (4) Fixed 0.5 m flying start distance (Fixed 0.5 m FD); and (5) Estimated flying start distance (Estimated FD) models, for both observed timing gate split times (black ●) and simulated timing gate split times (gray ◆). Simulated timing gate split times are generated using Laser estimates as a generative model, assuming a 0.5 m flying distance, and 0.01 s time rounding. Simulated timing gates models thus represent expected %MAD, given theoretical assumptions. Pooled data sets (i.e., Trial and Trial 2) were utilized. Gray bars represent a 5 and 10% MAD used as a visual anchor. Error bars represent 95% bias-corrected and accelerated (BCa) 5000 resamples bootstrap confidence intervals. Note: MSS—maximum sprinting speed (expressed in ms^−1^); TAU—relative acceleration (expressed in seconds); MAC—maximum acceleration (expressed in ms^−2^); PMAX—maximal relative power (expressed in W/kg).

Figure 11 depicts the estimated %MAD and accompanying 95% confidence intervals as error bars. Visual inspection of Figure 10 demonstrates that (1) only the No Correction model confidence intervals overlap for the simulated and observed data, while all other models demonstrated a higher %MAD than expected by simulation; and (2) only the MSS parameter demonstrated a %MAD below 5% for all models except for the No Correction and Fixed 0.5 m FD models, while all other parameters demonstrated a %MAD higher than 10.

The estimated percent minimum detectable change (%MDC_95_) using an agreement with the laser gun and pooled Trial 1 and Trial 2 data for the for the No Correction model ranged from 6.9 to 77.9%, for the Fixed +0.3 s TC model ranged from 12.9 to 64%, for the Estimated TC model ranged from 9.2 to 56.8%, for the Fixed 0.5 m FD model ranged from 16.9 to 87%, and for the Estimated FD model ranged from 11.2 to 80.1%.

The MSS parameter demonstrated the lowest %MDC_95_ across all timing gate models, ranging from 6.9 to 16.9%, while the TAU parameter demonstrated the highest %MDC_95_ ranging from 56.8 to 87%.

**Figure 12:**
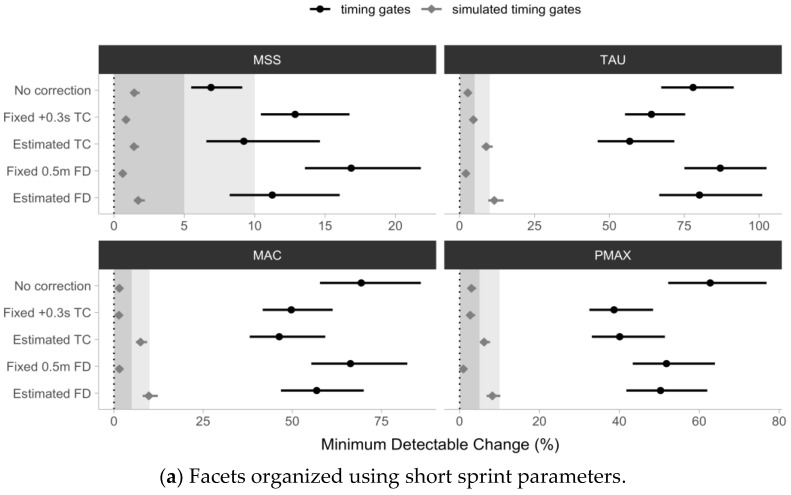

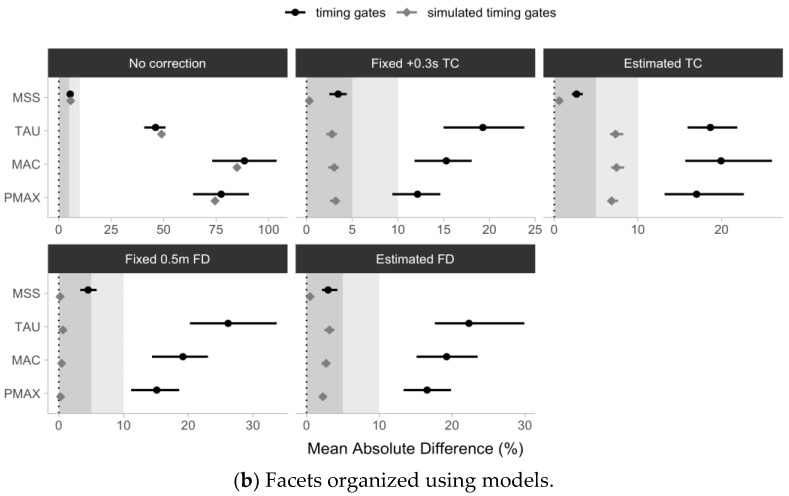
Estimated short sprint parameters minimal detectable change using the 95% confidence level ($\%MDCs_{95}$) for both observed timing gate split times (black ●) and the simulated timing gate split times (gray ◆). The method for estimating $\%MDCs_{95}$ utilized pooled Trial 1 and Trial 2 linear regression percent residual standard error (%RSE; Equation (13)) between the laser and (1) No Correction; (2) Fixed +0.3 s time correction (Fixed +0.3 s TC); (3) estimated time correction (Estimated TC); (4) fixed 0.5 m flying start distance (Fixed 0.5 m FD), and (5) estimated flying start distance (Estimated FD) models. Simulated timing gate split times are generated using laser estimates as a generative model, assuming a 0.5 m flying distance and 0.01 s time rounding. Simulated timing gates models represent the expected $\%MDCs_{95}$, given theoretical assumptions. Gray bars represent a 5 and 10$\%MDCs_{95}$ used as a visual anchor. Error bars represent 95% bias-corrected and accelerated (BCa) 5000 resamples bootstrap confidence intervals. Note: MSS—maximum sprinting speed (expressed in $ms^{-1}$); TAU—relative acceleration (expressed in seconds); MAC—maximum acceleration (expressed in $ms^{-2}$); PMAX—maximal relative power (expressed in W/kg).

Figure 12 depicts the estimated %MDC_95_ and accompanying 95% confidence intervals as error bars. Visual inspection of Figure 12 shows that (1) the %MDC_95_ was lowest for the MSS parameter, particularly the No Correction model, and that (2) all other parameters and models demonstrated a %MDC_95_ beyond what was expected by the simulated data.

## 4. Discussion

Valid and reliable estimation of the short sprint performance is one of the most important athlete profiling components [47,48]. The acceleration–velocity profile (AVP) (i.e., MSS and MAC parameters) represents a simple model to describe the kinematics of the short sprint performance. As such, it is attractive to sports practitioners to compare, evaluate, track, and monitor athletes across time and training interventions. For example, Bond et al. [47,48] have found that a single-beam infrared photocell and single-beam laser with a microprocessor demonstrated a considerably higher typical error and higher smallest worthwhile difference (i.e., %MDC_95_) when compared to a digital video camera as a preferred method for short-sprint performance assessment. However, in most instances, laboratory tools like the previously mentioned 3D motion cameras, videos, or laser guns are not readily available in all but a small number of elite sports teams. Thus, practitioners have been using split times measured using photocell timing gates to estimate maximum acceleration and maximum speed indicators. The recent development of the AVP model aimed to simplify this pursuit by consolidating various split time analyses into a simple and intuitive two-parameter model, where the MAC parameter represents an indicator of maximum acceleration characteristics and the MSS parameter represents an indicator of the maximum sprinting speed characteristic [4,5].

The present investigation revealed that agreement between laser gun and timing gates estimates using the percent bias (%Bias, or percent mean difference) estimator demonstrated the expected results. This was evident using the confidence intervals of the simulated timing gates and observed data being overlapping or touching for all models (apart from the Estimated TC model for the MAC and PMAX parameters) (Figure 10). Using the confidence intervals to judge statistical significance (i.e., with confidence intervals not crossing the zero line or other magnitude thresholds; [43]), the No Correction model showed bias involved in all parameters when estimated using a laser gun as the criterion. The Estimated TC model also demonstrated a statistically significant bias for all parameters except MSS. All other models did not demonstrate a statistically significant bias involved when estimating parameters. These findings add to the results obtained in a recently published study focused on examining bias in estimated short sprint profiles using simulation [41]. It has been found that the No Correction model demonstrated a notable bias in estimating short sprint parameters, while Estimated TC revealed a negligible bias alongside a higher proportion of simulations inside the region of practical equivalence (i.e., magnitude interpretation of the difference) [41].

When examining the agreement estimated using the percent mean absolute difference (%MAD) estimator, the study revealed the expected results for the No Correction model, with overlapping confidence intervals for the simulated and observed data. Every other model demonstrated a higher value compared to the expected values using the simulated data. Of all parameters, only MSS demonstrated a high agreement between the laser gun and timing gates estimates, using the estimator (below 5% for all models except for the No Correction and Fixed 0.5 m FD models). All other parameters demonstrated an unsatisfying agreement with the laser gun (>10%) (Figure 11). Although implementing different analysis procedures, it should be noted that Tillaar et al. [49] obtained similar findings pertaining to the difference in the measurement agreement between the timing gates and a laser gun. When performing 30-m sprints with a group of amateur female handball players, the authors found that a correction of +0.21 s needs to be used to obtain correct mechanical properties when using timing gates as a testing modality.

In addition to correctly estimating the current values of the short sprint parameters, practitioners are probably more interested in sensitivity to detect changes across time. When using the agreement with the laser gun, the MSS parameter showed the highest sensitivity (i.e., lowest %MDC_95_), and interestingly, it was the highest for the No Correction model. All other parameters and models demonstrated an unsatisfying level of sensitivity, beyond what was expected by the simulated data set (Figure 12). The lowest %MDC_95_ for the MSS parameter estimated with the No Correction model might be due to the simplest model utilized, and hence the reduced variance in the estimated parameters.

Overall, the results of the present investigation question the validity, reliability, as well as sensitivity of the AVP, estimated using timing gates, even with the novel correction models that were introduced. The maximum acceleration indicator (i.e., MAC) demonstrated a low agreement when compared to the laser gun, as well as unsatisfactory sensitivity to detect changes. The maximum sprinting speed indicator (i.e., MSS) demonstrated much better agreement with the laser gun and a satisfactory sensitivity to detect changes. Interestingly, the results indicated that the simplest No Correction model demonstrated the highest sensitivity to detect changes in MSS across all other timing gate models, although showing significant bias. Thus, practitioners should be wary of using timing gates to estimate maximum acceleration traits and changes in their respective levels.

While providing a deeper insight into the effects of the flying start on estimated short sprint profiles using a timing gate system, this study is not without limitations. The study utilized only one starting distance (i.e., 0.5 m from the initial timing gate) and each participant performed only two sprint trials in a single day. However, this method is ecologically valid, since it is the most common method of measuring short sprint performance by practitioners in team sports [34,35,36,37]. Future work should involve a similar study conducted with multiple sprints performed with different starting distances (i.e., on line, 0.5, and 1 m from the initial timing gate), positions (i.e., standing versus three-point or block start), triggering devices (foot pod, hand pod, etc.), types of timing gates (single-beam and double-beam photocells, single-beam laser with a microprocessor, etc.), and different levels of athletes, performed against the laser gun or 3D motion capture system over multiple days. Of particular interest, which is lacking in the current study, would be the assessment of between-days minimum detectable change, where multiple sprints would be repeated on non-consecutive days. This work would provide more insight into the most valid, reliable, and sensitive method of estimating the acceleration–velocity profile of the short sprints.

In conclusion, given the results of this study, practitioners using timing gates to estimate short sprint acceleration–velocity profiles in general, or maximum acceleration indices in particular, should be wary of using the results in judging the current state or performance improvement over time. Although maximum sprinting speed indices demonstrated satisfactory agreement and sensitivity, if interested in measuring and tracking maximum acceleration indices, researchers and practitioners should be cautious when using timing gates and should probably invest in more precise and sensitive technology, such as the laser gun, or perform video analysis [47,48].

## Figures and Tables

**Table 1:** Measured split sprint times by utilizing timing gates positioned at 5, 10, 20, 30, and 40 m.

Distance (m)	Split Time (s)
5	1.40
10	2.13
20	3.35
30	4.46
40	5.54

**Table 2:** Estimated sprint parameters for Andrew, Ben, and Cole. All three siblings exhibit equivalent sprint performance. However, they employ distinct sprint initiation techniques, leading to variations in split durations and, thus, divergent estimations of sprint parameters. The precision of the timing gates, which is accurate to two decimal places (i.e., 10 ms), results in estimated parameters for Andrew that deviate from the true values. MSS—maximum sprinting speed (expressed in ms^−1^). TAU—relative acceleration (expressed in seconds); MAC—maximum acceleration (expressed in ms^−2^); PMAX—maximal relative power (expressed in W/kg).

Athlete	MSS	TAU	MAC	PMAX
True	9.50	1.36	7.00	16.6
Andrew (theoretical)	9.54	1.37	6.96	16.6
Ben (flying start)	8.90	0.73	12.15	27.0
Cole (gunfire)	10.36	1.96	5.27	13.7

**Table 3:** Estimated sprint parameters for Andrew, Ben, and Cole using (1) No Correction; (2) Fixed +0.3 s time correction (Fixed +0.3 s TC); (3) Fixed +0.5 s time correction (Fixed +0.5 s TC); (4) Estimated time correction (Estimated TC); (5) Fixed 0.5 m flying start distance (Fixed 0.5 m FD); and (6) Estimated flying start distance (Estimated FD) models. Numbers in the brackets indicate the absolute percent difference from the true parameter value. For easier visual comprehension, absolute percent differences are depicted separately in Figure 8. Note: MSS—maximum sprinting speed (expressed in ms^−1^); TAU—relative acceleration (expressed in seconds); MAC—maximum acceleration (expressed in ms^−2^); PMAX—maximal relative power (expressed in Wkg^−1^); TC—time correction; FD—flying distance.

Model	Athlete	MSS	TAU	MAC	PMAX	TC	FD
True	True	9.5 (0%)	1.36 (0%)	7 (0%)	16.62 (0%)		
No Correction	Andrew (theoretical)	9.54 (0.4%)	1.37 (1%)	6.96 (0.6%)	16.6 (0.2%)		
No Correction	Ben (flying start)	8.9 (6.3%)	0.73 (46%)	12.15 (73.5%)	27.02 (62.5%)		
No Correction	Cole (gunfire)	10.36 (9%)	1.96 (44.8%)	5.27 (24.7%)	13.66 (17.9%)		
Fixed +0.3 s TC	Andrew (theoretical)	10.97 (15.5%)	2.37 (74.3%)	4.64 (33.7%)	12.72 (23.5%)		
Fixed +0.3 s TC	Ben (flying start)	9.51 (0.1%)	1.31 (3.2%)	7.24 (3.5%)	17.22 (3.6%)		
Fixed +0.3 s TC	Cole (gunfire)	12.92 (36%)	3.55 (161.4%)	3.64 (48%)	11.76 (29.2%)		
Fixed +0.5 s TC	Andrew (theoretical)	12.92 (36%)	3.55 (161.4%)	3.64 (48%)	11.76 (29.2%)		
Fixed +0.5 s TC	Ben (flying start)	10.29 (8.3%)	1.88 (38.8%)	5.46 (22%)	14.05 (15.5%)		
Fixed +0.5 s TC	Cole (gunfire)	16.99 (78.8%)	5.85 (331.1%)	2.9 (58.5%)	12.33 (25.8%)		
Estimated TC	Andrew (theoretical)	9.56 (0.6%)	1.38 (2%)	6.9 (1.4%)	16.5 (0.8%)	−0.01	
Estimated TC	Ben (flying start)	9.5 (0%)	1.3 (4.1%)	7.3 (4.3%)	17.33 (4.2%)	−0.30	
Estimated TC	Cole (gunfire)	9.56 (0.6%)	1.38 (2%)	6.9 (1.4%)	16.5 (0.8%)	0.19	
Fixed 0.5 m FD	Andrew (theoretical)	11.75 (23.7%)	2.96 (118.2%)	3.97 (43.3%)	11.66 (29.8%)		
Fixed 0.5 m FD	Ben (flying start)	9.52 (0.2%)	1.36 (0.5%)	6.98 (0.3%)	16.61 (0.1%)		
Fixed 0.5 m FD	Cole (gunfire)	15.83 (66.7%)	5.41 (299%)	2.92 (58.2%)	11.58 (30.4%)		
Estimated FD	Andrew (theoretical)	9.56 (0.6%)	1.38 (2%)	6.9 (1.4%)	16.5 (0.8%)		0.00
Estimated FD	Ben (flying start)	9.56 (0.6%)	1.4 (3.1%)	6.83 (2.4%)	16.31 (1.9%)		0.54
Estimated FD	Cole (gunfire)	9.06 (4.7%)	1.42 (4.3%)	6.4 (8.6%)	14.49 (12.8%)		0.00

**Table 4:** Final number of athletes in each trial used in the analysis for (1) the laser; (2) No Correction; (3) Fixed +0.3 s time correction (Fixed +0.3 s TC); (4) Estimated time correction (Estimated TC); (5) Fixed 0.5 m flying start distance (Fixed 0.5 m FD); and (6) Estimated flying start distance (Estimated FD) models.

Model	Trial 1	Trial 2	Trial 2-1
Laser	27	15	15
No correction	27	15	15
Fixed +0.3 s TC	27	15	15
Estimated TC	27	15	15
Fixed 0.5 m FD	27	15	15
Estimated FD	25	15	15

## Data Availability

The data and the R code can be found at https://osf.io/9v43e/ (DOI: 10.17605/OSF.IO/9V43E (15 April 2024)).
